# Report on Vaccine Farms

**Published:** 1882-05-20

**Authors:** James Law


### REPORT ON VACCINE FARMS.

BY DR. JAMES LAW.

The main objects in raising vaccine lymph on the lower animal are: 1st, to secure a sufficient supply to meet any sudden emergency; and 2d, to guard against the conveyance of disease by inoculation. The first of these objects is easily accomplished by the vaccine farms, and demands no remark from us. With regard to the second, it is justly claimed that the risk of the conveyance of the most objectionable of all maladies—syphilis—is entirely obviated by the use of bovine lymph. The authorities on vaccination claim, and doubtless with perfect justice, that the use of the pure vaccine lymph, even from a syphilitic person, cannot communicate the venerial disease, and that the danger only arises when blood has been mingled with the lymph or when the specific syphilitic action has begun in the sore. Yet the perfect exclusion of blood, or of blood serum, from the preserved lymph cannot be easily guaranteed in all cases, and the few instances of actual syphilitic infection from vaccination is an unanswerable argument for the use of bovine lymph.

Attention is therefore especially called to those diseases which may by any possibility be conveyed to the human being by the use of bovine lymph. These may be set down shortly as: Simple infecting inflammation, erysipelas, septic infection, malignant pustule, epizootic eczema, and perhaps, tuberculosis.

The frequent occurrence of local inflammation, of indolent inflammatory neoplasms, and of erysipelas around the seat of inoculation with bovine lymph, is a fact too notorious to be denied. It is true, that any one of these may be due rather to the state of the system inoculcated than to any fault in the lymph used. The germs of or the aptitude for a particular disease is present, and the occasion of its eruption is the irritation consequent on the inoculation, and it may be assumed that any other cause of an equivalent amount of local or constitutional disorder would have had the same result. When, however, the number of untoward cases mount up to one-fourth or one-half of the persons inoculated, and when simple wounds from other causes are attended by no such manifestations, it is only reasonable to conclude that the erysipelas or other morbid process has been determined by the lymph. Now, the normal products of the ox are productive of no such evil results. Butchers who are perpetually handling the carcasses of these animals are not specially subject to erysipelas around sores and abrasions. On the other hand, in cases in which the vaccinated calves had the surface operated on exposed to filth and wet, we have seen the resulting inflammation extend widely and prove very persistent and troublesome, virtually furnishing in the lower animal the counterpart of those abnormal results of vaccination which are occasionally seen in man. In view of this, too much care cannot be given to secure perfectly healthy, vigorous calves; to keep them in a pure, sweet atmosphere; to sedulously avoid all contact of the inoculated surface with decomposing manure and fæces, and to avoid taking lymph from any vesicle which has been already opened. Crusts are manifestly more objectionable than quills or points that have been charged from newly-opened vesicles. The boiling and thorough purification of quills and points before charging, and the prompt drying after having been charged, are absolutely essential to a guarantee of purity. The seclusion of the quills and points from flies and other insects before, but especially after, they have been charged is another obvious precaution. Not only will the insects speedily abstract all the virus, but, coming as they often do from putrid animal or vegetable matter or even from diseased bodies, they are liable to deposit other germs than those with which the objects have been charged. It is all-important to have the calves tied in seperate stalls, containing an abundance of clean litter, and the protection of the inoculated parts of the skin with a canvass cover is to be commended. It seems even as if the exposure of the lower surface of the body to pressure and friction when in the recumbent position, and its liability to contact with urine and fæces, would sanction a departure from the current mode, and the vaccination of the calf along the dorsal rather than the ventral surface. The objection that he would lick the surface operated on is easily met by applying beads around the neck, as is usually done in blistered horses. If a smaller supply of lymph is obtained than is obtainable from the looser skin below, it is a sufficient justification to say that our object is purity and not quantity. Finally, the farmer's objection that any resulting frizzling of the hair along the back deteriorates the animal in value goes for nothing when it is a question of the protection of man.

The taking of the virus early seems to be a very essential condition of securing it pure. Dr. Griffiths assures us that up to the seventh day the unbroken vesicle contains no superadded products, and will convey cow-pox uncomplicated. Later, when granular and pus cells appear, and above all when the reputed vesicle becomes the seat of septic and other bacteria, the virus cannot be sent with the same confidence. If, as Palasciano and others claim, the intensity of the virus is greatest from the third to the seventh day, and if at the same time its purity can be augmented in any way that cannot be accorded to it at a later stage of the eruption, the mere fact of a larger yield cannot sustain the practice of collecting the virus at a more advanced stage. The lessened supply may enhance the cost of the virus, and the method may necessitate the National Board of Health raising its own virus, yet it should not be a valid argument for the sanction of an article the purity of which is not guaranteed by every possible precaution.

*Epizootic eczema* (*the foot-and-mouth disease*) is not known to exist in the United States at present, yet it was imported from Europe as lately as a year ago, and as it has been repeatedly imported before, it is not impossible that it may visit us again. This is perhaps the most contagious disease known, and being propagated with equal readiness by all cloven-footed animals, it would be exceedingly likely to be introduced into any vaccine farm situated in a district where it had gained a footing. Chauvian has shown that cow-pox and epizootic eczema do not naturally exclude each other; they may exist together or successively in the same individual, and though it may be inocculated on any part of the skin or mucous membrane, epizootic eczema is especially liable to appear on the udder and inner sides of the thigh, the usual seat of vaccination in the calf. Again, this disease will develop in forty-eight hours after the reception of the germ, so that if introduced into the vaccinating stable in calves drawn according to the present system from a wide district, it may attack the animals already inocnlated, and the eruption of the two diseases will take place simultaneously. Probably not one of the directors of our vaccine establishments would recognize this disease if he were to see it; and if the lymph were clarelessly collected, it is just possible that the virus of this disease might be sent out. It need only be added that man is liable to the attacks of this malady, as well as are the lower animals.

*Malignant anthrax* has never been known to be conveyed by vaccination, but inasmuch as cattle are subject to this disease, and young cattle particularly so, as the morbid process is at times localized in the skin, and as it is communicable to man through the blood, there appears to be a possibility, though a remote one, of the communication of malignant pustule in vaccination. This should forbid the locating of vaccine establishments on or near marshes, drying-up ponds or lakes, rich river-bottoms, deltas, and the like, in which the anthrax germs, once deposited, are liable to be preserved for years, without losing their virulence. For the same reason heavy clays and other impervious soils are to be discarded in favor of sandy, gravelly, or other soils of a porous character. This preference to be given to open soils implies, further, the desirablility of breeding the calves on the vaccine farm in place of having them picked up without regard to locality, or a wide district or in public market by an unprofessional man.

The question of the possible conveyance of tuberculosis through bovine lymph is a very delicate one to approach. We have no evidence that tuberculosis has ever been conveyed in this way. There is, further, the consideration that a severe attack of cow-pox, like one of small-pox, is liable to be followed in a weak or strumous constitution by a scrofulous or tuberculous attack. This, however, is due to the disturbing and debilitating action of the disease rousing into activity the taint which was already present, and not the implanting of the poison along with that of variola. But it must be added that calves with a weak or strumous habit of body, or a hereditary predisposition to tuberculosis, are just as liable as human beings to develop that disease under the debilitating action of the cow-pox. It must be admitted also that among the calves selected for vaccination a preference is given to those of the Jersey breed, which in my experience is more subject to consumption than any other race of cattle in America. With small, delicate frames and an enormous capacity for the production of cream, this breed of cattle have had their powers of milk secretion stimulated and solicited until in many herds one-half have become the victims of tuberculosis. It must be conceded that, in a subject in which there is a strong tendency to tuberculosis, anything which establishes a local inflammation is especially liable to induce the tuberculous deposit, and that in the vaccinated calves the cutaneous irritation leads to congestion and enlargement of the glands of the flank and inguinal region which are especially liable to become the seat of tuberculosis in cattle. Certain it is that in some of the calves which had passed through vaccination I found these glands permanently enlarged, together with fever and other symptoms of tuberculosis.

Whether the tuberculous taint formerly present in the system or roused into activity by the operation of vaccination may be communicable through the lymph derived from the vesicles or through the blood accidentally mixed with the lymph, must be decided by absolute experiment. Meanwhile the prejudice that exists in the minds of a portion of the public on this question should demand that until this can be decided positively in the negative, the supposed danger should be guarded against by the use of calves the progeny of sound and vigorous animals only. This would necessarily do away with the present practice of using picked-up calves, and would demand a large breeding herd of carefully-selected animals, under the supervision of an accomplished veterinarian, whose duty it would be to detect and discard all animals which showed the slightest tendency to tuberculosis or other affection communicable to man.—*Nat. Board of Health Bulletin.*

